# Qualitative Approach to Attempted Suicide by Adolescents and Young Adults: The (Neglected) Role of Revenge

**DOI:** 10.1371/journal.pone.0096716

**Published:** 2014-05-06

**Authors:** Massimiliano Orri, Matteo Paduanello, Jonathan Lachal, Bruno Falissard, Jordan Sibeoni, Anne Revah-Levy

**Affiliations:** 1 INSERM 669 research unit, Paris-Sud University and Paris-Descartes University, Paris, France; 2 Department Applied Psychology, University of Padua, Padua, Italy; 3 Maison de Solenn, AP-HP Cochin Hospital, Paris, France; 4 Centre de Soins Psychothérapeutiques de Transition pour Adolescents, Argenteuil Hospital Centre, Argenteuil, France; University of Stirling, United Kingdom

## Abstract

**Background:**

Suicide by adolescents and young adults is a major public health concern, and repetition of self-harm is an important risk factor for future suicide attempts.

**Objective:**

Our purpose is to explore the perspective of adolescents directly involved in suicidal acts.

**Methods:**

Qualitative study involving 16 purposively selected adolescents (sex ratio1∶1) from 3 different centers. Half had been involved in repeated suicidal acts, and the other half only one. Data were gathered through semistructured interviews and analyzed according to Interpretative Phenomenological Analysis.

**Results:**

We found five main themes, organized in two superordinate themes. The first theme (individual dimensions of the suicide attempt) describes the issues and explanations that the adolescents saw as related to themselves; it includes the subthemes: (1) negative emotions toward the self and individual impasse, and (2) the need for some control over their lives. The second main theme (relational dimensions of attempted suicide) describes issues that adolescents mentioned that were related to others and includes three subthemes: (3) perceived impasse in interpersonal relationships, (4) communication, and (5) revenge.

**Conclusions:**

Adolescents involved in suicidal behavior are stuck in both an individual and a relational impasse from which there is no exit and no apparent way to reach the other. Revenge can bridge this gap and thus transforms personal distress into a relational matter. This powerful emotion has been neglected by both clinicians and researchers.

## Introduction

Adolescent suicide is a major public health concern in all western countries. Epidemiological data show that it is one of the three leading causes of death worldwide among those younger than 25 years [1], [2]. A more statistically widespread phenomenon is attempted suicide: its prevalence is about 7.8% in the United States [2] and 10.5% in Europe [3]. The highest attempted suicide rate is recorded among those aged 15–24 years, and their attempted/completed suicide ratio is estimated to be between 50∶1 and 100∶1 [4]. The prevention of suicidal behavior is therefore a primary social and medical concern throughout the world [1], [5]. Nonetheless, despite a large number of research and prevention programs, the attempted suicide rate among youth is increasing [6], and secondary prevention interventions have thus far achieved limited results [7], [8]. The numerous studies, conducted from multiple perspectives (including psychological, psychiatric, and sociological), show that one of the most important risk factors for attempted suicide is a previous attempt [9]–[11]. According to a recent English study, repetition of self-harm occurs in about 27% of adolescents, and the four major risk factors for repetition are age, prior psychiatric treatment, self-cutting, and previous self-harm. This study also found that youths who sought care at a hospital for self-harm are 10 times more likely to die by suicide than would be expected in this age group [12].

Although an understanding of the adolescent perspective is essential in preventing the relapse of suicidal behaviors, the subjective experience of those directly involved in suicidal acts has not been sufficiently explored [13]. Qualitative methods are particularly suited to investigating participants' viewpoints, their lived experiences, and their interior worlds [14], [15]. Nevertheless, qualitative research in adolescent suicidology is rare [16]. To our knowledge, only two qualitative studies [17], [18] have directly addressed the problem of relapse of suicidal or self-harming behavior among youth. In particular, one of them showed that current services respond inadequately to self-harming behaviors among young people and struggle to deal with the needs this population experiences [18].

The aim of this qualitative study is to explore the perspective of adolescents (for clarity's sake, we refer to our participants as adolescents) who have directly engaged in suicidal acts (in either single or repeated suicide attempts). Exploring the factors related to their success or failure in overcoming and moving beyond the suicidal period might provide clinicians with important insights useful in caring for young people involved in suicidal behavior, especially in a perspective of preventing repetition.

## Methods

### Participants and Setting

Participants received complete written information about the scope of the research, the identity and affiliation of the researchers, the possibility of withdrawing from the study at any point, confidentiality, and all other information required in accordance with Italian policies for psychological research and with the Helsinki Declaration, as revised in 1989. Participants (and their parents, for minors) provided written consent. This research received approval from the institutional review boards of the three hospitals involved: Santa Giuliana Hospital, Verona; Este Hospital, Padua; Monselice Hospital, Padua. These were two local general hospitals (with inpatient and outpatient adolescent psychiatric departments) and one psychiatric hospital in northeastern Italy. Physicians or psychologists at these hospitals were contacted and asked if they had patients who might be appropriate subjects for a study of adolescent suicide attempts. Subjects were eligible only if they had attempted suicide during adolescence or in the postadolescent period and were aged 15 to 25 years old at the time of the interview. Eligible subjects were then contacted.

Purposive sampling [19] was undertaken, and inclusion of subjects continued until saturation was reached [20]. As recommended for Interpretive Phenomenological Analysis (IPA) [21], [22], we chose to focus on only a few cases and to analyze their accounts in depth. Moreover, to include a heterogeneous sample with maximum variation [19], we included both adolescents with only a single suicidal act and those with multiple acts. We were therefore able to consider a wide range of situations and experiences. Sixteen Italian adolescents (sex ratio 1∶1) freely agreed to participate in the study (two refused, one male and one female). Their median age was 20 years at the interview, and 16 at the suicide attempt. Half had a history of previous attempts (≥1, see Table 1).

**Table 1:** Participants' characteristics.

Name	Gender (male/female)	Age at the interview (y)	Age at (first) suicidal act (y)	Repeated suicidal act (yes/no)
M1	male	18	16	no
M2	male	21	17	no
M3	male	19	17	no
M4	male	20	16	no
M5	male	20	18	no
M6	male	20	16	yes
M7	male	18	16	no
M8	male	19	16	yes
F1	female	17	16	no
F2	female	25	15	no
F3	female	18	17	no
F4	female	20	19	yes
F5	female	18	16	yes
F6	female	20	19	no
F7	female	24	15	yes
F8	female	20	19	yes

### Data Collection

Data were collected through 16 individual semi-structured face-to-face interviews. The interviews were audio-recorded and subsequently transcribed verbatim, with all nuances of the participants' expression recorded. An interview topic guide (Table 2) was developed in advance and included 8 open-ended questions and several prompts. The logic underpinning the construction of the interview guide was to elicit in-depth and detailed accounts of the subjects' feelings before the suicide attempt and afterwards, as well as the expectations and meanings that they connected to this action. Our overall objective in using this qualitative method was to put ourselves in the lived world of each participant and explore the meaning of the experience to each of them. Fourteen interviews took place at the adolescents' treatment facility, one at the adolescent's home, and one at the residential facility where the adolescent was living. Since the sensitive topic of our interviews, considerable attention was given to the evaluation of participants' opinion about the interview after its end. All adolescents felt comfortable discussing their experience and explaining their perspective without receiving any judgment from the researcher. Referent psychologists or physicians never reported any concern. In addition, researchers themselves discussed their own feelings about the interviews during study group meetings, in order to take into account potential influences on data collection and analysis (reflexivity).

**Table 2:** Interview topic guide.

Questions and prompts
1. What do you remember about the episode that led you to this emergency [suicidal act]? *Possible prompts*: how did you feel? What was your state of mind?
2. Let us talk about the previous period…Can you tell me something about your family? *Possible prompts*: what about your family life? Can you tell me more about the relationship with…?
3. Can you tell me something about your friends? *Possible prompts*: how do you feel within your peer group? Can you tell me more about the relationship with…?
4. Can you describe your wishes about the future?
5. After your suicide attempt, when you realize what happened, how do you feel? *Possible prompts*: can you tell me more about the moment when you met…?
6. What kind of changes there were in your life? *Possible prompts*: in your family life? Between your friends? How do you react to these changes?
7. What has it changed for you today?
8. When you made that decision [to attempt suicide], what did you think would happen? *Possible prompts*: what did you think people would understand?
Possible general prompts: Can you tell me more about that? How did you feel? Can you recall a particular example of that?

### Data Analysis

Qualitative analysis was performed according to IPA methodology. The aim of this method is to understand how people make sense of their major life experiences by adopting an “insider perspective” [23]. Three epistemological points underpin IPA: first, it is a phenomenological method that seeks to explore the informants' views of the world. As Husserl pointed out [24], the objective of phenomenology is to understand how a phenomenon appears in the individual's conscious experience. Hence, experience is conceived as uniquely perspectival, embodied, and situated [21]. Second, IPA is based on hermeneutics: interpretative activity, as defined by Smith & Osborn [22], is a dual process in which the “researcher is trying to make sense of the participant trying to make sense of what is happening to them”. In practice, during the analysis, the researcher might move dialectically between the whole and the parts, as well as between understanding and interpretation. Third, the idiographic approach emphasizes a deep understanding of the individual cases. IPA is committed to understanding the way in which participants understand particular phenomena from their perspective and in their context [21].

The analytic process proceeded through several stages: we began by reading and rereading the entirety of each interview, to familiarize ourselves with the participant's expressive style and to obtain an overall impression. We took initial notes that corresponded to the fundamental units of meaning. At this stage, the notes were descriptive and used the participants' own words; particular attention was paid to linguistic details, including the use of expressions (especially youth slang) and metaphors. Then conceptual/psychological notes were drafted, through processes of condensation, comparison, and abstracting the initial notes. Connections with notes were mapped and synthesized, and emergent themes developed. Each interview was separately analyzed in this way and then compared to enable us to cluster themes into superordinate categories. Through this process, the analysis moved through different interpretative levels, from more descriptive stages to more interpretative ones; every concept not supported by data was eliminated. The primary concern for researchers is to maintain the link between their conceptual organization and the participants' words [25]. For this reason, the categories of analysis are not worked out in advance, but are derived inductively from the empirical data.

To ensure validity, two researchers (MO and MP, both expert psychologists trained in qualitative research) conducted separate analyses of these interviews and compared them afterwards. A third researcher (ARL, psychiatrist specialist in qualitative research) triangulated the analysis. Every discrepancy was negotiated during study group meetings, and the final organization emerged from the work in concert of all the researchers. We agreed to considered data saturation to be reached because no new aspects emerged from the interviews (i.e. no more coded were added to our codebook) in each of our themes, and last interviews did not provide additional understanding of our participants' experience.

We report the study according to the COREQ statement. (Table S1)

## Results

**Figure 1:**
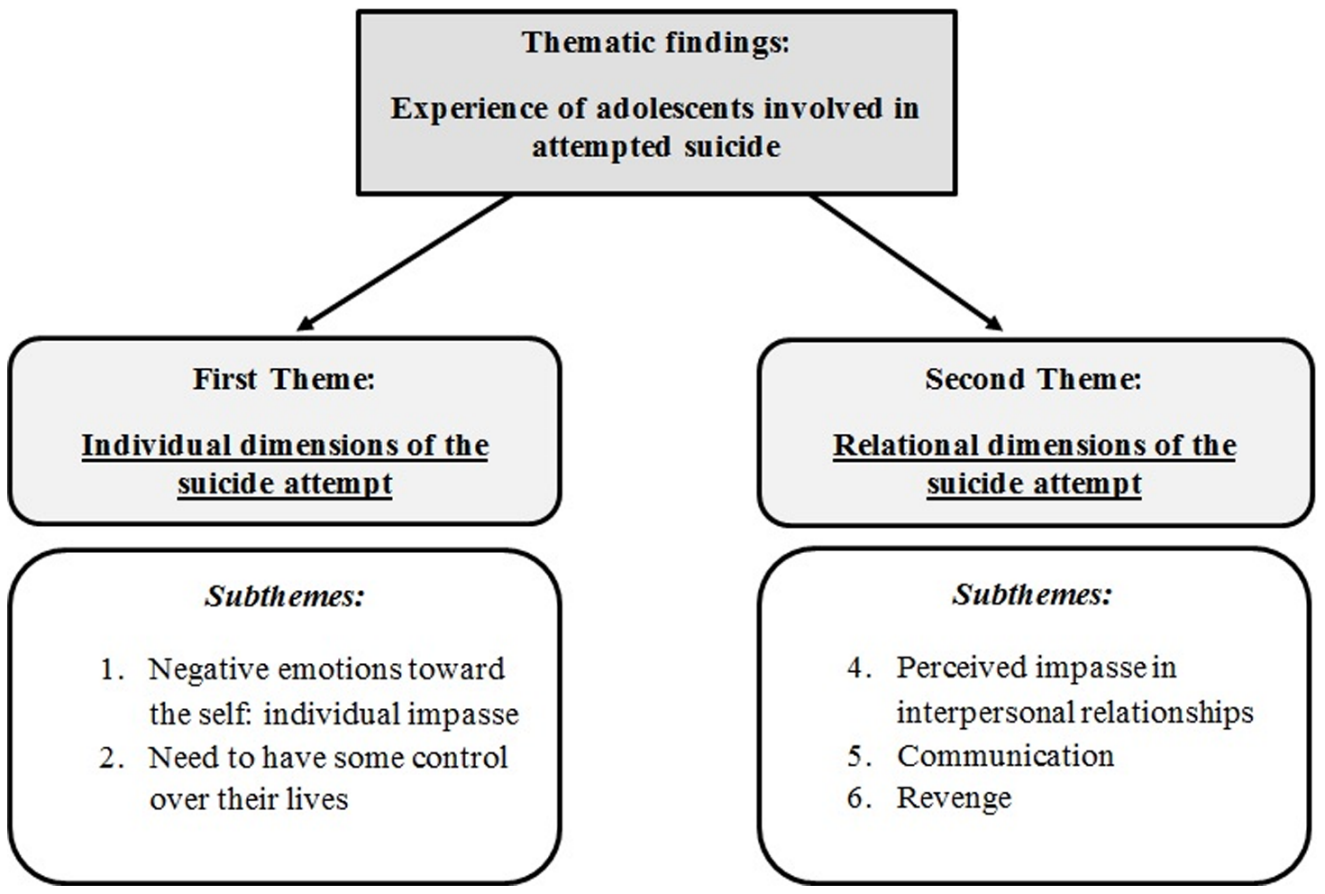
Thematic findings. Representation of themes and subthemes emerged from our analysis.

We identified five themes describing the experience of attempted suicide as narrated by participants and organized them into two superordinate themes, according to the meaning the adolescents attributed to their suicidal act (Figure 1): the first superordinate theme (Individual dimensions of the suicidal act) comprises the issues and explanations that the adolescents saw as related to themselves; it includes the themes: (1) negative emotions toward the self: the experience of an impasse with no exit, and (2) need to have some control over their lives. The second superordinate theme (relational dimensions of the suicidal act) involves issues with others in the three subthemes: (3) perceived impasse in family and peer relationships, (4) communication, and (5) revenge.

### Theme I: Individual dimensions of the suicide attempt

Two subthemes comprised this first theme: (i) negative emotions toward the self: the experience of an impasse with no exit, and (ii) the need to have some control over their lives.

1. **Negative emotions toward the self: individual impasse.** During the interviews all participants gave detailed descriptions of themselves, their state of mind, and the thoughts that led to the decision to attempt suicide. The words they used to talk about themselves described a devalued self, in which their dominant feeling was that they were not accepted.
  That day, I took the pills looking myself in the mirror…I kept repeating that I was disgusting, that no one really cared about me…[I was thinking] that everything about me was wrong! That nothing I did came out right…I don't know, I continued this thing of not feeling accepted, not feeling that anybody cared about me… (F4).
  Shame and guilt were the feelings that adolescents evoked most frequently during the interviews, and their narratives were dominated by a sense of estrangement, loneliness, and loss of any meaning to their lives. One participant described her feelings of loneliness with a meaningful metaphor:
  I was alone, stretched out on the ground, I didn't know what to hang on to…I was looking in vain for something to hang on to, but I failed…essentially I was alone… (F3).
  From almost every adolescent's account emerged the feeling of trapped in a suffering present, with no better future possible. They described feeling as if they were in a blind alley, had no more energy, and were completely surrounded, vanquished; they felt it was impossible to find a viable alternative to get out of their situation and give their life a different meaning. One girl's question bluntly demonstrated the disintegration of the meaning of her life: *“what am I doing in this life?” (F2)*:
  I thought to myself: ‘what am I doing in this life?’…I didn't accept myself, I wasn't accepted by my family and…so, I was depressed, I was depressed in that period, that's for sure…because for me it was really finished…I wanted to finish it, I'd had enough (F2).
  The suicidal act appeared salvational, a way to free oneself from an intolerable condition. Participants thus used positive adjectives to describe what they were seeking (air, light, freedom), expressing the hope that their act would lead them out of the impasse in which they felt trapped.
  I only saw blackness around me, and perhaps those [suicide attempts], they were the only white things I could see… I wanted to see the light. I was convinced that if I died I would see white, light…a light bulb turning on…it was a conviction I had. Because I saw everything black, always darkness…between the black that I saw [that others created around me] and the black I created around me, I thought that dying…you know, all these attempts, I wanted to see the light…you know, to breath… (F8).
2. **Need to have some control over their lives.** These adolescents broached issues of control and mastery during their interviews in several ways. During the period before their act, they lived a situation that they perceived was out of their control. They described their struggles to move beyond this lived situation that, as we have just reported, appeared impossible to overcome or resolve, that they experienced passively, were subjected to. What emerged from the interviews was that acting on their body offered them control of/over their life, in contrast to all the other uncontrollable situations they were living. Half of the adolescents interviewed had cut themselves as a positive action, to make themselves the actor of something in their life.
  I had no control over the others, but I had control over myself…so I could do what I wanted to myself …and the cuts were a way to comfort my pain… I still have the scars – blood everywhere, I was crying, but…but the problem was still there…however, during these moments […] it was as if I had control of my life… (F7).
  These adolescents lived their suicide attempt as an escape from an overwhelming life situation that was beyond their ability to manage:
  I said ‘that's OK, stop, let's finish it off, that way, I'll put everything straight…I won't have to think about anything anymore, there won't be anything to deal with, and…everything will be better.
*Interviewer: What do you mean by “everything will be better”?*
  That is, more than anything, that there will be nothing else so it will necessarily be better! […] I was glad to have made that decision… I was glad and sure about my decision… (M7).
  Narratives related to the post-suicidal period shed light on the failure of the adolescents' attempts to achieve control of their own lives. They talked about feeling of anger, described as a physical and violent rage closely linked to the failure of their act, and about finding themselves in a situation they perceived as still more difficult. They lived the failure of their act as yet another demonstration of their ineptitude, just one more in their long string of personal failures.
*Interviewer: What about the changes in your life [after the suicide attempt]?*
  Nothing…maybe, I began to see things darker […], I thought I wasn't able to do anything, that I was afraid…now I'm tired, I can't take it anymore, before it wasn't like this […]. I began to see everything as darker…I began to think that I was wrong, that I was the problem…because when there is a problem now, I give up…and before it wasn't so. From that, I feel my life has changed (F6).

### Theme II: Relational dimensions of the suicide attempt

The second superordinate theme is the relational dimension of the suicidal act. The three subthemes belonging to this domain are described below:

1. **Perceived impasse in interpersonal relationships.** Our participants' narratives of their family relationships focused on the description of an impasse, a sort of gridlock dominated by the absence of acceptance or trust and the perception of being written down or even off. It seems to parallel the negative emotions toward the self and the perceived impasse described above (theme 1).
  Because I was changing and they didn't realize that, they only realized it when I ran away from home […] at the beginning, I did it because…that is, I didn't even think about it much, but then, as the hours were going by I kept on thinking about it and…I don't know, but it was like running away to make myself visible… (M1).
  The participants described rigid and overwhelming family dynamics and their perception that it was impossible to escape an unbearable situation. They also directly linked their need to escape and their choice to attempt suicide:
  “When I began to make her understand that I wasn't going to accept this situation anymore, all hell broke loose…and then, from that, my act…since I began to tell her ‘look, Mama, I can't take that anymore.’ …she didn't accept that…maybe she understood I'm no longer the baby who's happy with a new pair of shoes so she'll be good, keep quiet and make believe she's happy…I don't know…”
*Interviewer: can you tell me more about the relationship between that and your act?*
  I think that it is… the fundamental relationship…I think that is the main reason that I did it, fundamentally… (F6).
  The peer group was also described as a source of intense emotions. Although the narratives revealed that the teens hoped their peer group might supply what their families failed to give them, these texts also demonstrated fragility. Sometimes, they felt that being part of their peer group produced emotions very like to those about their family life; this increased the feelings of loneliness and of not being understood:
  I felt they were superficial, and I didn't want to keep on pretending to be like that…I didn't feel at ease with them, and slowly I lost the people I went out with (M5).
  A frequent topic was the emotional investment in one core relationship, an investment the adolescents perceived as a way to cope with the instability and difficulties of their lives. It was described in terms of dependency: the relationship became the repository of their hopes, and the person they were involved with, the reference point of their life:
  My ex-boyfriend F. was my first one…I was sixteen…my first sexual relationship, my first love story, it lasted 3 and a half years. He was my reference, because my parents are separated, my father is far away, and I have an awful relationship with my mother…and he was like… like an older brother… a father…his mother was like a mother to me, and she was almost my mother for three and a half year […]. With F. I had finally found that kind of stability…but, I guess it was only a stopgap, a stopgap that covered up all my problems…and in fact, when he was gone, they all reappeared on the surface (F3).
2. **Communication.** All the participants explicitly described the communicative issues related to their suicide attempt. It is clear that each suicidal act was primarily an interpersonal act, concerning not only the self but also the environment of significant others. The suicide attempt was closely linked to a situation with which the adolescent could not deal — all efforts were in vain. Suicide thus became the only possible way to get the person to listen to the adolescent's difficulties and to send a message that was impossible to deliver otherwise. The suicidal act was described as the only choice, once every other communicative possibility had failed.
  I was sick and tired of my mother's behavior…and to keep on talking was useless. I went on for several months and kept talking and talking and…that was hurting me…and I was tired. And so I finally did something like that [attempted suicide], but it was mainly to make her understand that she was killing me!…either she would kill me, or…or I had to find another way […]. If I tried to do that there, it's because I had already talked about it in every other way… (F4).
  Our analysis of the narratives about the period after the suicidal act found these youth travelled two different paths. Those who successfully emerged from the suicidal crisis described the first as a progressive opening of the line of communication with others, a process that established a basis for a change in the family relationship:
  Before, I didn't even talk to her, while now we can talk fairly peacefully…about school, or work…that kind of thing…we always spoke about my past, and we each understood … what she felt and what I felt…yeah, we talked about that […] I told her what I had been doing in [that town], what I was doing, what substances I was taking…and she told me she was always crying, that she was desperate, always worrying, that she had done everything possible to make me come back home again […] So, I realized that I had made her suffer so much, and that she had done so much for me…to help me, but I didn't realize that… I wasn't going to listen to her, or even give a damn,… because I believed that she couldn't possibly succeed in understanding me… (F1).
  This excerpt shows that the communication that developed after the suicide attempt led to the explicit recognition of feelings, emotions, and thoughts that had been present before the attempt, but never successfully communicated. It is important to note that it was not a dialogue about the suicidal act, but an attempt at mutual understanding.
  The second path was that of the adolescents who described a situation in which dialogue and communication remained as impossible after the suicide attempt as it has been before. The communication so unambiguously embedded in the attempt remained unanswered. The indifference described by the participants — including, for some, their family's refusal to admit they had attempted suicide — had the effect of reinforcing the feelings that led to the attempt.
  They didn't create a good situation…they act like they did when I crashed the car when I was drunk… They rub it in that they can't even fall asleep at night, they rub everything in, they were really full of hatred…and every time I did it [attempted suicide], it was always worse, because they were increasingly irritated, and I increasingly hated them…and so…the situation just kept getting worse (F7).
3. **Revenge.** A strong relational theme that the participants described explicitly was revenge. Several adolescents explained the aggressiveness of their act as a way to make other people feel guilty for their deaths and made the vindictive intent of the attempted suicide very plain, as the following excerpt shows:
  I was convinced, utterly convinced…yeah, yeah, I want to do it…revenge! Revenge!
*Interviewer: can you explain to me a little better, revenge? What were you thinking?*
  So, it means…this is what you've gotten by behaving like this to me all these years…you've gotten only my hatred, my contempt…my contempt for life…and…and now you look at me…look at me and suffer (F5).
  Revenge carries a message, one intended to make the others aware of their mistakes, their carelessness. One adolescent described it as a communication that was impossible to misunderstand: finding her body will cause her parents “*suffering*, *crying, and regret*” (*F5*). It almost appears that she expects to be present to witness the scene. It is a way to put the blame on others and make them feel guilty through remorse:
  I can't fully understand why I did it [attempted suicide]…but if I think about it, I honestly would say that I did it to punish her [my mother]…to pay her back… (F4).
  I wanted to die, I wanted to die to make my ex feel guilty, to make my parents feel guilty, that they hadn't known how to listen to me when I needed… I also wanted to make others feel guilty…I wanted to die, yes, because I was suffering, but at the same time I wanted to make them feel guilty … make them feel like shit…I wanted to make them cry, I wanted to destroy their lives…(F5).
  …I've thought a lot about it…several days or weeks before, I was thinking, but not about how to do it or what to do…but only how to revenge myself on them. They made me suffer so much and now they were sitting around calmly, as if nothing had happened…all right. They had ruined my life and did not even realize it. So, I had no other choice…to make them understand (M4).

## Discussion

Our phenomenological analysis of young adults' accounts of their suicide attempts elicited five themes that described the experiences they lived. These themes were organized into two superordinate themes, according to whether they concerned the individual or the relational dimensions that emerged from the narratives. We showed that the attempts to link the two dimensions — to communicate their anguish —were a key aspect of our participants' experience. The vengeful meaning of suicide that we found exemplifies this attempt to reach a relational dimension, to hurt someone else by hurting oneself. Accordingly to Knoll [26], revenge is an intense and pervasive emotion that has nevertheless received little attention, especially in the domain of youthful suicidal behavior. Our findings showed that revenge is a strong other-directed emotion, which aims to communicate an individual's own internal state by inflicting permanent suffering on others — by suicide. This revenge, moreover, is not only directed at other but is also a means of relieving one's own intense experience of internal struggle and helplessness.

Clinicians caring for suicidal adolescents need to acknowledge the violence (aggressiveness and revenge) inherent in the suicidal act. It is not obvious for them to think about violence, aggression, and revenge when they are confronted with these teens. This study provides an opportunity to illuminate this aspect of suicide and make clinicians aware of the role of this powerful emotion. We argue that openly addressing this issue with adolescents themselves and their families may play an essential role helping them recognize the multiple factors (both individual and relational, as we showed) that led to a particular suicide attempt, to put things in perspective (clarifying the individual/relational confusion), and begin the process of moving beyond the crisis and avoiding a repetition.

### Comparison with the literature

Our findings are consistent with previous work. The subthemes of the first theme (individual dimension of attempted suicide) show the subjective experience of loneliness, isolation, and negative emotions toward the self. The experience of suicidal acts described by adolescents is primarily a solitary experience involving the loss of any meaning in life and the impossibility of finding another way to exit a perceived impasse. Studies focusing on the internal world of the suicidal adolescent have consistently demonstrated negative emotional experiences [17], [27], [28]. We show that the need to recover control over one's own life plays an important role in the decision to kill oneself, as others have found [9],[18],[28] for people involved in non-suicidal self-harming behaviors [29].

The subthemes of the second theme deal with the relational dimensions of the act. Adolescents described the meaning of the situation that led to their decision to attempt suicide with interpersonal explanations, such as a lack of communication with their family and peers, a sense of not belonging to either group, and the impossibility they felt of overcoming an interpersonal stalemate. Moreover, they recounted changes that the primary suicidal act produced (or failed to produce) in their interpersonal world that eventually enabled important relationships to be restructured in ways that, for example, increased mutual understanding. Several authors have investigated the relational aspects of suicide attempts in various populations, including LGBT [30], ethnic minorities [31], and depressed adolescents [32]. Consistently with our findings, these studies pointed out the importance of interpersonal relations in understanding both the reasons for suicide attempts and the patterns of recovery in adolescent suicidal behavior.

We go further, however. Although previous studies have mentioned the relation between the individual and interpersonal dimensions of suicidal acts, they have not discussed it clearly, and several gaps remain. The hypothesis we propose, which emerges from our findings, is that confusion exists between these two dimensions. Adolescents continually try to link their individual state of personal distress, helplessness, and loneliness to the presence of others, seeking to connect. They described situations in which their unhappiness is not recognized or acknowledged by others. Our findings suggest that for adolescents suicidal behavior represents a means of establishing a connection between their personal distress and the others, through the act itself. Revenge, as discussed above, is one way to do that.

Moreover, failure to establish that link appears to be a major factor responsible for keeping the adolescent in the same state of mind that led to the initial act and thus keeps him or her at risk for repeating it.

### Limitations

This study has two main limitations. The first concerns its generalizability. Our purposive sampling procedure allowed us to include a wide sample of experiences among young men and women, with both single and multiple suicidal acts, of different durations of time since the act, and initially treated at 3 different hospitals. Nonetheless, our findings can be generalized only to young Italian adults, and attitudes may differ in other countries or even in other regions of Italy. However, our methodological precautions assure the trustworthiness of our findings. Because the socio-cultural environment has a strong influence on suicidal behaviors [31], further research needs to be conducted to compare and integrate perspectives from several countries. The second limitation is that all the participants were contacted through a healthcare facility where they underwent a period of psychiatric or psychological treatment. This might have affected the way that they retrospectively understood their act

### Conclusion and perspectives for future research

Adolescent suicidal behavior appears to be a relational act that aims to bridge a gap between the adolescents and their significant others in order to resolve a perceived impasse. Failure – by the others and by the therapist – to recognize this intent and take it into account appears to be a key factor for repetition of this behavior. Revenge assumes a particular role that appears to have been neglected by both clinicians and researchers until now, and further research should address this issue. Additionally, qualitative studies should be conducted to understand both caregivers' and health-care professionals' perspectives about the issue of revenge in adolescent suicide attempts.

## Supporting Information

Table S1 COREQ checklist.
(DOCX)
