# Supplementary material for: Qualitative Approach to Attempted Suicide by Adolescents and Young Adults: The (Neglected) Role of Revenge
Source: PLoS One. 2014 May 6;9(5):e96716. doi: 10.1371/journal.pone.0096716 (PMC4011950; doi:10.1371/journal.pone.0096716)
Supplement: Table S1 — COREQ checklist. (DOCX) [file pone.0096716.s001.docx]

**Table S1. COREQ checklist (Consolidated criteria for reporting qualitative research)**[1]**.**

| No Item | Guide questions/description | Page (explanation) |
| --- | --- | --- |
| **Domain 1: Research team and reflexivity** | | |
| **Personal Characteristics** |  |  |
| 1. Interviewer/facilitator | Which author/s conducted the interview or focus group? | 6 |
| 2. Credentials | What were the researcher’s credentials? | 1 (title page) |
| 3. Occupation | What was their occupation at the time of the study? | 1 (title page) |
| 4. Gender | Was the researcher male or female? | Both male |
| 5. Experience and training | What experience or training did the researcher have? | 6 |
| **Relationship with participants** |  |  |
| 6. Relationship established | Was a relationship established prior to study commencement? | Any relationship |
| 7. Participant knowledge of the interviewer. | What did the participants know about the researcher? e.g. personal goals, reasons for doing the research | 4 (Participants were full informed about the scope of the research; according to ethical policies) |
| 8. Interviewer characteristics | What characteristics were reported about the interviewer/facilitator? | 6 |
| **Domain 2: study design** | | |
| **Theoretical framework** |  |  |
| 9. Methodological orientation and Theory | What methodological orientation was stated to underpin the study? | 5-6 |
| **Participant selection** |  |  |
| 10. Sampling | How were participants selected? | 4 |
| 11. Method of approach | How were participants approached? | 4 |
| 12. Sample size | How many participants were in the study? | 4 |
| 13. Non-participation | How many people refused to participate or dropped out? Reasons? | 5 |
| **Setting** |  |  |
| 14. Setting of data collection | Where was the data collected? | 5 |
| 15. Presence of non-participants | Was anyone else present besides the participants and researchers? | No |
| 16. Description of sample | What are the important characteristics of the sample? | 4-5, Table 1 |
| **Data collection** |  |  |
| 17. Interview guide | Were questions, prompts, guides provided by the authors? Was it pilot tested? | 5, Box 1 |
| 18. Repeat interviews | Were repeat interviews carried out? If yes, how many? | No |
| 19. Audio/visual recording | Did the research use audio or visual recording to collect the data? | 5 |
| 20. Field notes | Were field notes made during and/or after the interview or focus group? | No |
| 21. Duration | What was the duration of the interviews or focus group? | 40 to100 min each |
| 22. Data saturation | Was data saturation discussed? | 4 |
| 23. Transcripts returned | Were transcripts returned to participants for comment and/or correction? | No |
| **Domain 3: analysis and findings** | | |
| **Data analysis** |  |  |
| 24. Number of data coders | How many data coders coded the data? | Not explicated |
| 25. Description of the coding tree | Did authors provide a description of the coding tree? | 5-6, Figure 1 |
| 26. Derivation of themes | Were themes identified in advance or derived from the data? | 6 |
| 27. Software | What software, if applicable, was used to manage the data? | No |
| 28. Participant checking | Did participants provide feedback on the findings? | No |
| **Reporting** |  |  |
| 29. Quotations presented | Were participant quotations presented to illustrate the themes / findings? Was each quotation identified? | 7-10 |
| 30. Data and findings consistent | Was there consistency between the data presented and the findings? | 7-10 |
| 31. Clarity of major themes | Were major themes clearly presented in the findings? | 7-10, Figure 1 |
| 32. Clarity of minor themes | Is there a description of diverse cases or discussion of minor themes? | 7-10, Figure 1 |

**Reference:**

1. Tong A, Sainsbury P, Craig J (2007) Consolidated criteria for reporting qualitative research (COREQ): a 32-item checklist for interviews and focus groups. Int J Qual Health Care 19: 349–357.
